# Isolated Hemihyperplasia in Adolescence: A Case Report

**DOI:** 10.7759/cureus.90189

**Published:** 2025-08-15

**Authors:** Roger Solano, Karina Gomez, David Solano, Magda Mendez, Nadia V Padilla-Claros, Sendy Ruiz-Zaldivar, César Alas-Pineda

**Affiliations:** 1 Pediatrics, Lincoln Hospital, New York, USA; 2 Faculty of Medicine and Surgery, Catholic University of Honduras, San Pedro Sula, HND; 3 Quantitative Biomedical Science Program, Dartmouth College, New Hampshire, USA

**Keywords:** adolescent, asymmetry, case report, congenital, hyperplasia

## Abstract

Isolated hemihyperplasia is a rare condition characterized by asymmetric overgrowth of the body that is not associated with overgrowth syndromes. We report the case of a 16-year-old adolescent with isolated congenital hemihyperplasia affecting the left hemibody, who had never undergone specific evaluation for this condition, as the clinical manifestations were mild and did not result in significant alterations beyond the observable asymmetry. Notable findings included slightly finer and thinner scalp hair texture on the contralateral side to the hemihyperplasia, as well as bilateral brachymetatarsia of the third toes. Clinical criteria and molecular testing were essential for establishing an accurate diagnosis.

Given the association between hemihyperplasia and tumor risk, screening for neoplasms using abdominal ultrasound and serum alpha-fetoprotein testing is recommended. This condition presents a clinical challenge, as coordinated intervention by specialists in orthopedics, nephrology, and genetics is required to ensure effective clinical management.

## Introduction

Hemihyperplasia, traditionally referred to as hemihypertrophy, is a condition that occurs in approximately 1 in 13,200 live births and is characterized by asymmetric overgrowth on one side of the body, affecting areas such as the face, trunk, limbs, digits, or internal organs, or involving the entire half of the body [1-3]. It may appear as an isolated finding or be associated with overgrowth syndromes, including Beckwith-Wiedemann syndrome, Klippel-Trenaunay-Weber syndrome, Proteus syndrome, Russell-Silver syndrome, and neurofibromatosis type I [2,4].

The isolated form of hemihyperplasia is rarer than the form associated with overgrowth syndromes, which may lead to misdiagnosis and the oversight of the associated neoplastic predisposition [5]. The accurate diagnosis of a patient with hemihyperplasia is based on clinical criteria and molecular tests that help distinguish between isolated cases and those associated with syndromes, which enables a more targeted and effective medical approach [1,5,6].

The cause of isolated hemihyperplasia remains unknown, and therefore, its diagnosis is made by exclusion [2]. Due to its similarity with Beckwith-Wiedemann syndrome, it is suggested that it may share the same etiology [1,7,8]. This involves a genomic imprinting alteration in the 11p15 chromosomal region, a DNA segment involved in growth regulation [1,7,8]. This genetic modification leads to abnormal expression of growth-regulating genes and, consequently, asymmetric body development [8]. It is important to note that this alteration accounts for 80% of the etiology of Beckwith-Wiedemann syndrome. However, in isolated hemihyperplasia, it has been documented in only a minority of cases [6,7]. Patients with isolated hemihyperplasia typically present with varying degrees of asymmetry, most commonly affecting the right side, and may exhibit vascular anomalies or scoliosis as a result of limb length discrepancy [2,5,7].

Tumor surveillance is of critical importance in patients with isolated hemihyperplasia, as a correlation has been identified, similar to that observed in syndromic cases, between the affected side and a predisposition to neoplasms such as hepatoblastoma, Wilms tumor (with a general risk of <5% in cases of isolated hemihyperplasia), leiomyosarcoma, or adrenal cortical carcinoma [2,4,7]. This case report presents a 16-year-old male patient with congenital isolated hemihyperplasia, characterized by left-sided asymmetry involving the thorax, kidney, and both upper and lower extremities. Prominent venous distention was also noted on the affected side. Additional notable findings included bilateral brachymetatarsia of the third toes and slightly finer and thinner hair texture on the right side of the scalp. It highlights the importance of a comprehensive and multidisciplinary approach including orthopedic, genetic, nephrological, and psychological evaluations for effective clinical management, encompassing early and accurate diagnosis and personalized medical intervention.

## Case presentation

A 16-year-old male patient, with a known diagnosis of left-sided hemihyperplasia, presented as a new patient for a Well-Child Check. The patient reported having had physical asymmetry since birth; however, this had not been a cause for concern, as no alterations in growth, development, mobility, or intelligence were observed. He indicated that he did not regularly attend annual medical check-ups and had never received a specific evaluation for his condition, with the risk being underestimated. He reported being evaluated by orthopedics only once since childhood due to recurrent episodes of knee and hip pain after prolonged periods of exercise. He was diagnosed with flat feet, but no further follow-up was provided due to his subsequent relocation abroad. Informed consent was obtained from the patient for the publication of this case and any related images.

**Figure 1:**
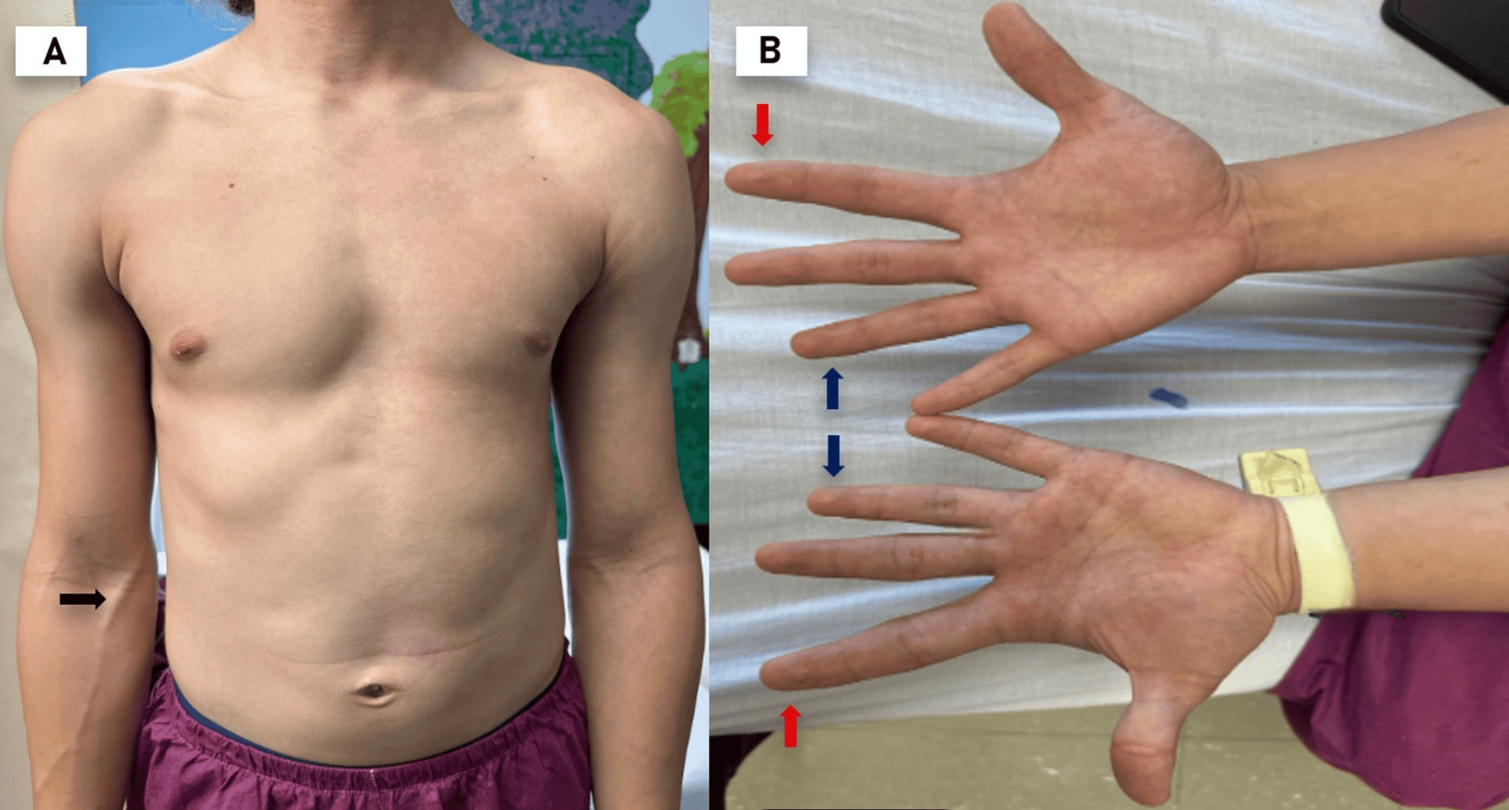
Physical examination (A) Asymmetry of the muscles on the left side, accompanied by increased visibility of the vein on the same side (black arrow), findings consistent with left-sided hemihyperplasia. (B) Hand and finger asymmetry is also observed. The left hand appears thicker and longer. The index finger is elongated (red arrow), while the ring finger appears shortened (blue arrow) on both hands.

During the physical examination, it was noted that the hair to the right of the midline was slightly finer and thinner, while the hair on the left side of the head had a normal texture. The left thorax appeared larger than the right. Heart sounds were normal, regular, and of a normal rhythm. There was no evidence of abnormal vascular sounds upon auscultation and palpation of the cranial region and other body areas. Marked venous distension was noted on the left side of the body (Figure 1A). The left upper and lower limbs, including the hands, were thicker and longer (Figure 1B).

**Figure 2:**
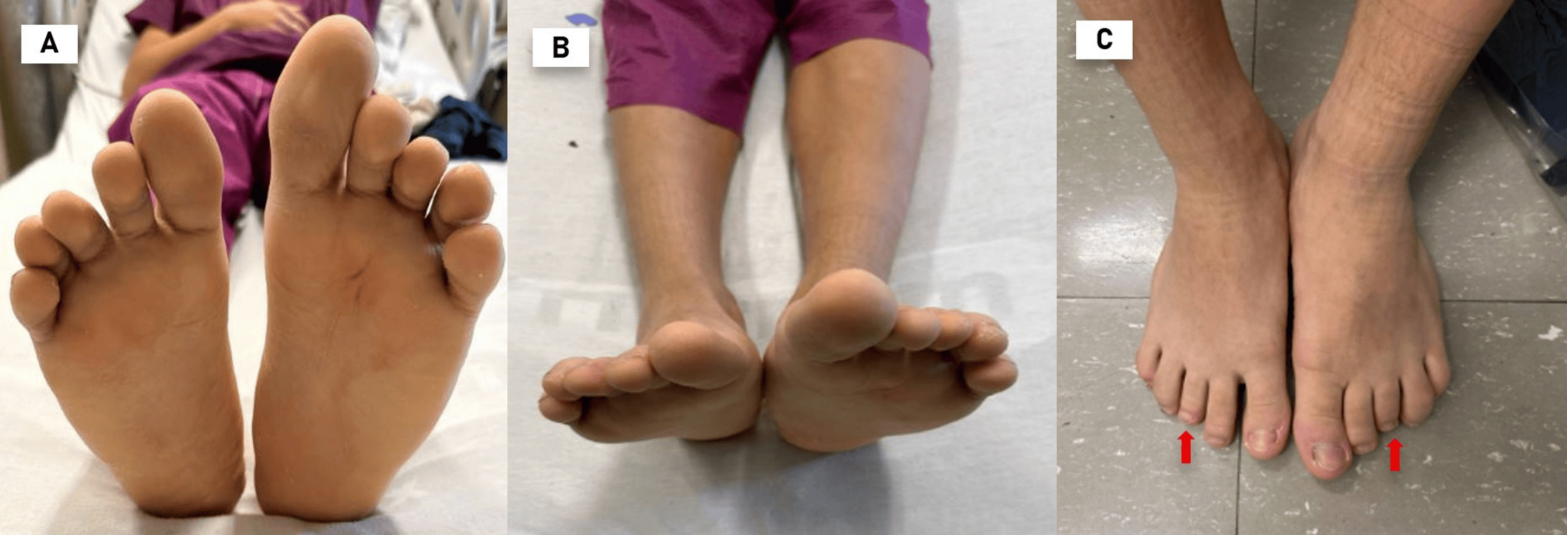
Lower limb assessment (A) Observable length discrepancy of 1.5 cm between the two lower limbs, indicative of underlying limb asymmetry. (B) Asymmetry in foot length, more pronounced on the left side, viewed from an overhead perspective. (C) Bilateral brachymetatarsia of the third toe (red arrow), a rare finding seen in isolated hemihyperplasia.

In the assessment of the lower extremities, the bone length scanogram revealed that the right lower limb measured 89.1 cm and the left 90.6 cm, indicating a 1.5 cm difference between the two sides, with no other significant findings (Figure 2A, Table 1). The patient was subsequently referred to podiatry, where a clinical method was employed to assess limb length, using the anterior superior iliac spine, located at the front of the pelvis, as the reference point, extending to the medial malleolus, situated distally on the tibia. The left limb measured 39 inches, while the right measured 38.5 inches (Figure 2B). Additionally, brachymetatarsia of the third digit was observed bilaterally (Figure 2C). Custom-made orthotic insoles with a polypropylene base and a 1/4-inch heel lift for the right foot were recommended.

**Table 1:** Body structure measurements Quantitative imaging data including bone length scanogram findings indicating a 1.5 cm discrepancy in lower limb length, with the left limb longer than the right, and renal ultrasound measurements consistent with mild enlargement of both kidneys.

Body Structure	Right (cm)	Left (cm)
Lower limb	89.1	90.6
Kidney	10.6 × 5.6 × 6.8	11.1 × 5.1 × 6.5

**Figure 3:**
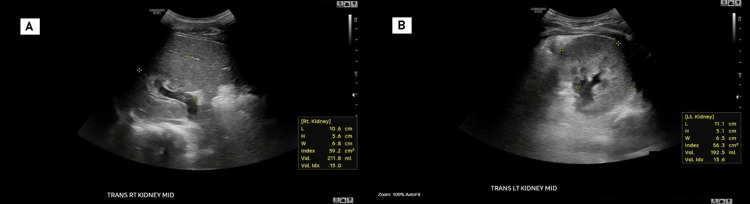
Renal ultrasound screening (A) Mild enlargement of the right kidney, with normal echotexture and cortical thickness. It measures 10.6 x 5.6 x 6.8 cm. No hydronephrosis, calculi, cysts, or solid masses are observed; (B) Mild enlargement of the left kidney, with normal echotexture and preserved cortical thickness. It measures 11.1 × 5.1 × 6.5 cm. Mild left-sided pelvicaliectasis is noted, which resolves following voiding, consistent with marked bladder distention. This finding excludes a pathological cause of pelvicaliectasis, such as hydronephrosis, a potential complication of Wilms tumor. No visible calculi are present. No cystic or solid masses are identified.

A tumor screening was performed using renal ultrasound and alpha-fetoprotein (AFP) testing as a measure of tertiary prevention, given that hyperplasia represents a risk factor for the patient. The renal ultrasound showed slightly enlarged kidneys, with normal echotexture and cortical thickness, without evidence of cysts or masses. The right kidney measured 10.6 x 5.6 x 6.8 cm (Figure 3A) and the left 11.1 x 5.1 x 6.5 cm (Figure 3B, Table 1). It is important to consider the slight enlargement of the kidney, as although it is not abnormal, its asymmetry coincides with the rest of the body, making this finding an important factor for monitoring due to the potential associated risks. On the other hand, the AFP tumor marker was within the normal range, with a value of 2.3 ng/mL (normal value ≤ 8.3 ng/mL).

Similarly, in order to identify the molecular basis of hemihyperplasia and to establish a differential diagnosis between isolated hemihyperplasia and that associated with overgrowth syndromes, the patient was referred to the genetics department. There, a high-resolution chromosomal microarray and a miscellaneous genetic analysis were performed to detect large-scale genomic alterations and submicroscopic variations, respectively. The chromosomal microarray interrogated approximately 2.67 million probes across the entire genome, including clinically relevant loci such as 11p15 (imprinting center regions 1 and 2), CDKN1C, and H19, among others related to the differential diagnosis of the suspected condition. In this patient, no pathogenic variants, regions of homozygosity, or uniparental disomy were identified. Both tests yielded negative results, thereby ruling out potential diagnoses of Beckwith-Wiedemann, Silver-Russell, Proteus, Simpson-Golabi-Behmel, and Sotos syndromes, and supporting the diagnosis of isolated hemihyperplasia (Table 2).

**Table 2:** Case findings in isolated hemihyperplasia Findings are consistent with left-sided isolated hemihyperplasia. A rare feature, brachymetatarsia, was also observed. Genetic testing showed no evidence of syndromic association. Tumor screening was performed with no abnormalities detected.

Case findings in isolated hemihyperplasia
Physical Examination
Left side findings: The thorax was larger, with increased venous distention. The upper and lower limbs were thicker and longer compared to the right.
Right side finding: Hair was slightly finer and thinner.
Rare finding: Bilateral brachymetatarsia of the third toes.
Renal Ultrasound
Mild bilateral enlargement of the kidneys.
Genetic Testing
High-resolution chromosomal microarray and miscellaneous genetic testing ruled out syndromic forms of hemihyperplasia.
Tumor Screening
No hydronephrosis, renal calculi, cysts, or solid masses were observed on renal ultrasound.
Alpha-fetoprotein levels were within the normal reference range (2.3ng/mL).

During one year of follow-up, the patient's condition remained stable, with no new significant findings in the physical examination. It is essential to continue with periodic clinical follow-up, including imaging surveillance for early detection of potential complications, such as scoliosis and the development of neoplasms. Furthermore, it is recommended to implement orthopedic treatment combined with psychological support, as part of a comprehensive strategy to address the patient's body asymmetry. This multidisciplinary approach aims to improve quality of life, support emotional well-being, and prevent future complications.

## Discussion

Hemihyperplasia, traditionally known as hemihypertrophy, is a rare disorder characterized by asymmetric enlargement of one or more body parts on one side, resulting from excessive cellular proliferation [2,9-11]. This overgrowth may also be accompanied by asymmetric visceromegaly [2].

This condition may also present as an isolated finding or in association with other clinical features as part of a group of syndromes commonly referred to as “overgrowth syndromes” [12]. These include Beckwith-Wiedemann, Sotos, Malan, Marshall-Smith, Weaver, Simpson-Golabi-Behmel, Perlman, Bannayan-Riley-Ruvalcaba, PI3K-related syndromes, Proteus, fibrous dysplasia, Klippel-Trenaunay-Weber, and Maffucci syndromes [6,13]. These syndromes are characterized by congenital malformations, abnormal growth patterns, and a frequent association with neoplastic processes [6].

Determining the precise prevalence of isolated hemihyperplasia remains a challenge, as many mild cases may go undiagnosed [3]. It is estimated to occur in approximately 1 in 13,200 live births [1]. However, this estimate may not be entirely accurate, as it includes both isolated and syndromic forms of the condition [13]. An international multicenter prospective study involving populations from Mexico, the United States, Australia, Israel, and Ukraine screened 10,066 patients and reported a higher incidence of 1 in 5,000 for isolated hemihyperplasia [9].

Cases have been documented in which a diagnosis of the disease was not established, with the clinical presentation being interpreted as swelling of unknown origin [11]. In other cases, adequate follow-up was not performed, nor was tumor screening conducted in patients with risk factors despite presenting this condition, as occurred in the described case, where the clinical manifestations were mild and did not seem to affect structures beyond the observable asymmetry [2]. Diagnostic errors have also been reported, where hemihyperplasia was incorrectly identified as another pathological entity, as observed in a patient with intestinal lymphangiectasia [14].

In the medical literature, similar cases of isolated hemihyperplasia of the left side have been documented, with manifestations such as renal and extremity enlargement, differences in contralateral hair texture, and the absence of cardiac abnormalities [15,16]. Vascular prominences have also been described in overgrowth syndromes associated with hemihyperplasia, such as in Klippel-Trenaunay-Weber syndrome [15]. Likewise, at least half of the patients with hemihyperplasia exhibit cutaneous abnormalities such as edema, hirsutism, hemangiomas, nevi, capillary dilation, or café-au-lait spots [6,13]. In cases of isolated hemihyperplasia, vascular lesions and capillary dilation have also been reported [15].

However, no reports were found in the reviewed literature describing the concomitant presence of bilateral brachymetatarsia of the third toe in cases of isolated hemihyperplasia. Brachymetatarsia, a rare congenital malformation predominantly observed in childhood and adolescence, can cause biomechanical disruptions and pain, which is why conservative treatment is recommended. It is an atypical presentation in isolated hemihyperplasia, as it has not been reported in this condition, although it does appear in association with other genetic syndromes.

The medical literature describes the association of brachymetatarsia with syndromic disorders, and within growth-related diseases, clinical manifestations such as fifth digit clinodactyly in Russell-Silver syndrome; polydactyly in the PI3K-related syndrome; camptodactyly in the Weaver syndrome; and, in Simpson-Golabi-Behmel syndrome, deformities such as metatarsus varus, short feet, cutaneous syndactyly, hypoplasia of the index nail, and postaxial polydactyly [6,17]. Furthermore, it is important to highlight the reported cases of isolated hemihyperplasia presenting macrodactyly [5].

The patient's clinical presentation suggested hemihyperplasia; however, it was the clinical criteria and genetic testing that enabled the differential diagnosis between the isolated form and that associated with syndromes [6,18]. Since the hemihyperplasia was not accompanied by key diagnostic signs of any overgrowth syndrome, and genetic testing was negative for such conditions, a diagnosis of isolated hemihyperplasia was made by exclusion [6,18].

The risk of patients with isolated hemihyperplasia developing embryonal tumors is well documented. Wilms tumor is the most common, but other tumors such as adrenocortical tumors, neuroblastomas, and sarcomas may also occur [2]. Hoyme et al. [2] conducted a 10-year prospective study in which they followed 168 children diagnosed with isolated hemihyperplasia. They found six cases of Wilms tumor, one hepatoblastoma, two adrenocortical carcinomas, and one leiomyosarcoma of the small intestine, which represented a tumor incidence of 5.9% [2].

A 3% to 5% risk of developing Wilms tumor has been identified in children with isolated hemihyperplasia or Beckwith-Wiedemann syndrome [19]. Consequently, due to the associated tumor risk, early screening for neoplasms is recommended, using abdominal ultrasounds (aimed at identifying renal tumors, particularly Wilms tumor) and serum AFP tests (targeted at detecting liver tumors, especially hepatoblastoma) [2,6,11,20].

Screening recommendations include monitoring children with isolated hemihyperplasia who present isolated hypermethylation of the H19 gene or paternal uniparental disomy of chromosome 11p15 [4]. It is suggested to perform renal ultrasounds every three months until the age of seven, complemented by physical evaluations every six months until adulthood [20]. This recommendation is based on the median age for diagnosing Wilms tumor, which is three and a half years; however, although less common, it can also manifest in adolescents and adults [9]. Furthermore, it is advised to measure serum AFP every three months until the age of four, as 90% of hepatoblastoma cases occur during this period [18].

A limitation of this case report is the lack of clinical evaluation of other family members to exclude asymmetries, considering that familial occurrences of isolated hemihyperplasia have been reported [1]. 

It is important to note that, although the risk of neoplasms decreases during adolescence, the presence of body asymmetries accompanied by internal anomalies may significantly affect the patient's quality of life [9]. In this context, it is recommended to complement the diagnostic approach with radiographic studies of the skeletal extremities and cranial computed tomography to rule out the involvement of bone structures and internal organs [10].

## Conclusions

Hemihyperplasia is a rare congenital condition that can cause significant body asymmetry and requires interdisciplinary management, posing challenges for both accurate diagnosis and effective treatment. It may present as an isolated finding or in association with various overgrowth syndromes. Genetic testing and established clinical criteria are key to diagnosing isolated hemihyperplasia. Regular follow-ups are essential to monitor growth and address orthopedic issues, while continuous surveillance for tumor development, particularly Wilms tumor, remains crucial due to the increased risk. Early detection of mild and asymptomatic cases is key to initiating appropriate care and preventing missed diagnoses. This comprehensive approach facilitates timely intervention and supports the best possible outcomes for the patient.

Furthermore, this report presents two findings to highlight in the context of isolated hemihyperplasia: a slightly finer and thinner scalp hair texture on the contralateral side to the hemihyperplasia, and a possible coexistence between isolated hemihyperplasia and brachymetatarsia, for which no reports were found in the reviewed literature.
